# Minocycline/Amoxicillin-Based Bismuth Quadruple Therapy for *Helicobacter pylori* Eradication: A Pilot Study

**DOI:** 10.3390/microorganisms12030429

**Published:** 2024-02-20

**Authors:** Senlin You, Xiaoqiong Tang, Jiarui Zhou, Yalin Shen, Xiaona Song, Mohammed Benghezal, Barry J. Marshall, Hong Tang, Hong Li

**Affiliations:** 1 West China Marshall Research Center for Infectious Diseases, Center of Infectious Diseases, West China Hospital, Sichuan University, Chengdu 610041, China; senlinyou_912918@126.com (S.Y.); hxtxq1992@163.com (X.T.); zhoujiarui0125@163.com (J.Z.); shenyalind@163.com (Y.S.); 18683725447@163.com (X.S.); mbenghezal@gmail.com (M.B.); barry.marshall@uwa.edu.au (B.J.M.); 2 Division of Infectious Diseases, State Key Laboratory of Biotherapy, West China Hospital, Sichuan University, Chengdu 610041, China; 3 *Helicobacter pylori* Research Laboratory, School of Biomedical Sciences, Marshall Centre for Infectious Disease Research and Training, University of Western Australia, Nedlands, WA 6006, Australia

**Keywords:** *Helicobacter pylori*, minocycline, antibiotic resistance, bismuth quadruple therapy

## Abstract

The common adverse effects and the complicated administration of tetracycline and metronidazole greatly affect the clinical application of the classical bismuth quadruple therapy (BQT) for *Helicobacter pylori* eradication. This pilot study aimed to evaluate the efficacy and safety of minocycline/amoxicillin-based BQT for *H. pylori* eradication. Firstly, consecutive *H. pylori* isolates collected at West China Hospital of Sichuan University between 2018 and 2021 were included for susceptibility testing of tetracycline and minocycline using E-test strips. Secondly, both treatment-naïve and experienced patients were included to receive a 14-day minocycline/amoxicillin-based BQT: esomeprazole 40 mg or vonoprazan 20 mg, bismuth colloidal pectin 300 mg, amoxicillin 1000 mg, and minocycline 100 mg, all given twice daily. Among a total of 101 *H. pylori* isolates, tetracycline resistance was 3.0%, whereas minocycline resistance was nil. A total of 114 patients (treatment-naïve/experienced, 72/42) received the minocycline/amoxicillin-based BQT. The overall intention-to-treat (ITT) and per protocol (PP) eradication rates were 94.7% (108/114) and 97.3% (108/111), respectively. The ITT and PP eradication rates were 91.7% (66/72) and 95.7% (66/69) among the treatment-naïve patients, and both were 100.0% among the treatment-experienced patients. No serious adverse event was recorded. This pilot study suggests that minocycline/amoxicillin-based BQT is an excellent therapy for *H. pylori* eradication.

## 1. Introduction

*Helicobacter pylori* is a gastric pathogen that infects approximately half of the world’s population [1]. Usually acquired in childhood, the infection persists for a lifetime without antibiotic treatment. Chronic *H. pylori* infection may progress to severe gastric disease including peptic ulcer, gastric cancer, and gastric mucosa-associated lymphoid tissue (MALT) lymphoma. It has been well-recognized that *H. pylori* infection is the most important risk factor for the development of gastric cancer as 89% of worldwide gastric cancer can be attributable to *H. pylori* infection [2]. Successful eradication of *H. pylori* heals peptic ulcers and effectively reduces the risk of gastric cancer [3]. However, with the worldwide increase in *H. pylori* resistance to clarithromycin, levofloxacin, and metronidazole, the efficacies of the legacy triple therapy regimens have decreased to unacceptably low levels [2,4]. The latest guidelines have recommended the classical bismuth quadruple therapy consisting of a proton pump inhibitor (PPI), bismuth, tetracycline, and metronidazole as the first-line or rescue therapies [2,4,5]. However, tetracycline is largely unavailable in many parts of the world, and the high frequency of adverse events and the complicated administration of tetracycline and metronidazole (three or four times a day) greatly affect the clinical feasibility of this classical bismuth quadruple therapy.

Minocycline, a second-generation and semisynthetic tetracycline derivative, is well absorbed after oral administration. It binds to the 30S subunit of the bacterial 70S ribosome, preventing bacterial protein synthesis, and resulting in a bacteriostatic effect [6]. Compared with other tetracyclines, minocycline has better bactericidal activity, a higher lipid affinity, and a longer half-life. In addition, due to its proven human safety data and broad antimicrobial spectrum, minocycline has been widely used in clinical practice for the treatment of many infectious diseases [7,8]. Previous studies have shown that minocycline had excellent in vitro bactericidal activity against clinical *H. pylori* isolates, and the in vitro minocycline resistance is rare [9,10,11]. Previous clinical studies have also evaluated the efficacy of minocycline-containing regimens as first-line or second-line therapies for *H. pylori* eradication [9,12,13]. However, conflicting results were reported with both satisfactory and unsatisfactory efficacies among different studies [9,12,13,14]. Of note, most of the minocycline-containing bismuth quadruple regimens were minocycline in combination with high-dose metronidazole (400 mg, four times a day) [11,13,14,15,16]. The use of high-dose metronidazole is for partially overcoming the alarmingly high worldwide metronidazole resistance. However, the higher the dose of metronidazole, the more frequent the occurrence of metronidazole-related side effects such as nausea, dizziness, and metallic taste. The inconvenient use of high-dose metronidazole (four times a day) and the subsequent common side effects substantially affect patient compliance. Thus, the metronidazole in the classical bismuth quadruple regimen may also need to be replaced with another antibiotic, which should be more convenient for administration and have low resistance.

Amoxicillin, a semisynthetic penicillin derivative, has been widely used for *H. pylori* eradication treatment. Through binding to the penicillin-binding protein 1A (PBP1A) located on the inner membrane of the bacterial cell wall, amoxicillin acts to interfere with bacterial cell wall synthesis, causing cell lysis and thus having a bactericidal activity. Of note, resistance to amoxicillin is negligible (less than 5%) in most of the countries [17]. Thus, for patients with *H. pylori* infection and without allergy to penicillin, amoxicillin is always the first choice in the design of antibiotic combination regimens for *H. pylori* eradication treatment. Amoxicillin is usually prescribed 1 g twice a day in both legacy triple therapy and bismuth quadruple therapy regimens, whereas 1 g three times a day is prescribed in high-dose dual therapy.

The combination of amoxicillin with minocycline for *H. pylori* eradication has only been reported in three previous studies [9,11,12]. Murakami et al. reported a 7-day triple therapy with amoxicillin (750 mg bid), minocycline (100 mg bid), and rabeprazole (20 mg bid), which only achieved a 38.5% eradication rate [9]. The poor efficacy may be partly ascribed to the short therapy duration and inadequate dose of amoxicillin. The second and third studies both conducted in China reported a 14-day bismuth quadruple therapy with amoxicillin (1000 mg bid), minocycline (100 mg bid), rabeprazole (10 mg bid), and bismuth potassium citrate (220 mg bid), which achieved ITT eradication rates of 87.5% [12] and 85.7% [11] for first-line therapy, respectively. The efficacy of this minocycline/amoxicillin-based bismuth quadruple therapy is not optimal, which is likely to be further improved by increasing the dose of PPI or replacing it with the potassium-competitive acid blocker, vonoprazan. Vonoprazan has a stronger and more sustained acid-inhibitory effect than existing PPIs. Sustained adequate intragastric acid suppression is crucial for maintaining the potency of antibiotics for successful *H. pylori* eradication.

The primary objective of this study was to clinically evaluate the safety and efficacy of a 14-day minocycline/amoxicillin-based bismuth quadruple therapy (with vonoprazan or double dose of PPI) for first-line and second-line *H. pylori* eradication. In addition, we also measured the in vitro antibacterial activity of minocycline against clinical *H. pylori* isolates previously collected at West China Marshall Research Center for Infectious Diseases, West China Hospital.

## 2. Materials and Methods

### 2.1. H. pylori Strains and Antibiotic Susceptibility Testing

A total of 101 clinical *H. pylori* strains previously collected and stored at −80 °C freezer at West China Hospital of Sichuan University between 2018 and 2021 were included. For antimicrobial susceptibility testing (AST), these frozen isolates were thawed and cultured on commercial Columbia blood agar (CBA) plates (diameter 90 mm, thickness 4 mm; Autobio, Zhengzhou, China). The plates were incubated at 37 °C in sealed jars with microaerophilic conditions (N_2_:H_2_:CO_2_, 85%:5%:10%) generated by the Anoxomat Mark-II system (Mart Microbiology B.V., Lichtenvoorde, The Netherlands). After 1–3 days of incubation, the recovered isolates were sub-cultured onto new CBA plates for approximately 24 h.

For antimicrobial susceptibility testing of tetracycline and minocycline, the freshly grown isolates were suspended in sterile saline at a density of 1.0 OD_600_ or approximately 5 × 10^8^ CFU/mL, and an inoculum of 100 μL of the bacterial suspension was spread evenly by a cotton swab on the surface of commercial CBA plate. The tetracycline and minocycline E-test strips (Liofilchem, Roseto degli Abruzzi, Italy) were then applied onto the surface of the inoculated CBA plates. After incubation for 3–5 days, the MICs were read at the point of intersection of the elliptical zone of growth inhibition with the MIC scale on the E-test strips. Based on the European Committee on Antibiotic Susceptibility Testing (EUCAST) recommendations, resistance to tetracycline was defined as minimum inhibitory concentration (MIC) > 1.0 mg/L. Resistance to minocycline was defined as MIC > 1.0 mg/L based on previous studies [9,10].

### 2.2. Patients

The evaluation of minocycline/amoxicillin-based quadruple therapy for *H. pylori* eradication was a prospective study conducted at West China Hospital of Sichuan University between January 2021 and August 2023. Both treatment-naïve and treatment-experienced patients with *H. pylori* infection determined by ^13^C-urea breath test (^13^C-UBT) (Shenzhen Zhonghe Headway Bio-Sci & Tech Co., Ltd., Shenzhen, China) were enrolled in this study. Patients with one of the following criteria were excluded: younger than 18 years old; taking any drugs that may influence the study result such as proton pump inhibitors (PPI), bismuth salts, and antibiotics within 4 weeks prior to enrollment; severe concomitant diseases such as severe liver and kidney dysfunction; history of allergy to any of the study drugs; and women who were pregnant or lactating. All participants signed the informed consent. The study was approved by the Ethics Committee of West China Hospital of Sichuan University (2017/332).

### 2.3. Minocycline/Amoxicillin-Based Quadruple Therapy

Patients were prescribed minocycline/amoxicillin-based quadruple therapy consisting of acid inhibitor (esomeprazole 40 mg or vonoprazan 20 mg), bismuth colloidal pectin 300 mg, amoxicillin 1000 mg, and minocycline 100 mg, all given twice daily for 14 days. Acid inhibitor and bismuth colloidal pectin were taken half an hour before meals. Amoxicillin and minocycline were administered half an hour after meals. We explained to all patients the importance of compliance with the treatment regimen to achieve successful eradication, as well as how to report potential adverse events during the treatment period.

### 2.4. Evaluation of Efficacy, Safety Profile, and Compliance

*H. pylori* infection status was assessed by ^13^C-UBT at least 4 weeks after the end of treatment. Successful eradication of *H. pylori* was considered when the ^13^C-UBT test was negative. Adverse events and compliance were recorded using questionnaire by patient self-report charts during the treatment period. Within 3 days after treatment, the questionnaire was collected to evaluate the incidence of adverse events and therapeutic compliance. Adverse events were classified as mild (causing discomfort but not interfering with daily activities), moderate (partially affecting daily activities), and severe (causing considerable interference with daily activities). Good compliance was defined as taking ≥ 80% of the prescribed drugs.

### 2.5. Statistical Analysis

The statistical analysis was performed using SPSS (version 19, Chicago, IL, USA). Continuous variables were described as the mean ± standard errors of the mean (SEM), and categorical variables were expressed as numbers or percentages and compared by Pearson’s χ^2^ or Fisher’s exact test. The eradication rates with minocycline-based quadruple therapy were calculated using intention-to-treat (ITT; including all eligible participants who took at least one dose of medicine regardless of their compliance with the study protocol) and per protocol (PP; including patients who fully followed the protocol and excluding those with poor compliance or loss of follow-up) analysis. *p* < 0.05 was considered statistically significant.

## 3. Results

### 3.1. Antimicrobial Activity of Minocycline and Tetracycline against 101 Isolated Clinical H. pylori Strains

**Figure 1:**
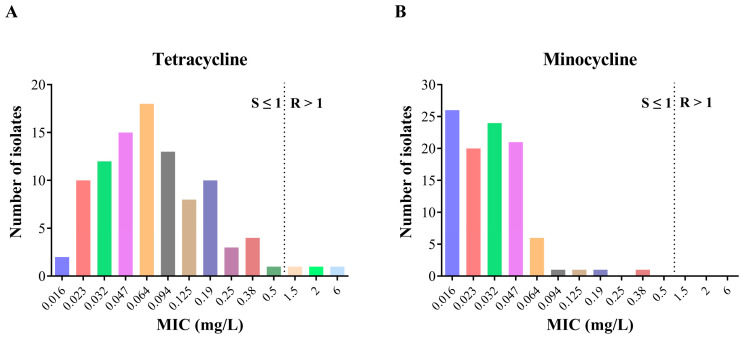
Susceptibility patterns of tetracycline and minocycline in the 101 *H. pylori* strains. (**A**) MIC distribution for tetracycline; (**B**) MIC distribution for minocycline. The dashed lines indicate the resistance cut-off values for tetracycline and minocycline. MIC, minimum inhibitory concentration.

To compare the in vitro antibacterial activity of minocycline and tetracycline against *H. pylori*, the MICs of minocycline and tetracycline among 101 clinical *H. pylori* strains (Table S1) isolated at West China Hospital between 2018 and 2021 were simultaneously determined using the E-test strips. We demonstrated that the antibiotic resistance rate to tetracycline was 3.0% (3/101), whereas no isolate was found to be resistant to minocycline (Figure 1).

The MICs against tetracycline and minocycline were both low. However, compared with tetracycline having a wide MIC distribution (24 strains had MICs ≤ 0.032 mg/L, 46 strains had MICs in the range of 0.047–0.094 mg/L, 26 strains had MICs in the range of 0.125–0.5 mg/L, and the remaining 3 tetracycline-resistant strains had MIC at 1.5, 2, and 6 mg/L, respectively), the minocycline MICs were mostly ≤0.032 mg/L (*n* = 70). In addition, there were 21 strains having minocycline MIC at 0.047 mg/L, 6 strains at 0.064 mg/L, and the remaining 4 strains at 0.094 mg/L, 0.125 mg/L, 0.19 mg/L, and 0.38 mg/L, respectively (Figure 1). Of note, of the three tetracycline-resistant strains, strain 55# had tetracycline MIC = 6 mg/L and minocycline MIC = 0.023 mg/L; strain 68# had tetracycline MIC = 1.5 mg/L and minocycline MIC = 0.38 mg/L; and strain 82# had tetracycline MIC = 2 mg/L and minocycline MIC = 0.19 mg/L (Table S1). Although the three tetracycline-resistant strains remain susceptible to minocycline, cross-resistance between the two structurally related antibiotics might be a concern for clinicians, as it can be reversely seen that the two strains with the highest minocycline MICs (0.38- and 0.19 mg/L) were both tetracycline-resistant.

Taken together, our results revealed that minocycline had stronger in vitro antibacterial activity against *H. pylori* as compared with that of tetracycline. Tetracycline resistance was negligible, and no minocycline resistance was found among the 101 clinical *H. pylori* isolates.

### 3.2. Evaluation of the Efficacy, Safety, and Compliance of the Minocycline-Based Quadruple Therapy

A total of 114 (treatment-naïve/experienced, 72/42) consecutive patients were enrolled with a mean age of 48.8 years (Table 1). Among the included patients, 62 were prescribed with EMAB regimen (esomeprazole, bismuth colloidal pectin, amoxicillin, and minocycline), and 52 patients were prescribed with VMAB regimen (vonoprazan, bismuth colloidal pectin, amoxicillin, and minocycline). Of note, among the 72 treatment-naïve patients, 3 patients (all receiving EMAB regimen) were excluded for PP analysis (1 patient discontinued treatment due to COVID-19 infection, 1 patient lost to follow-up, and 1 patient had no ^13^C-UBT taken after eradication treatment). Of the 42 treatment-experienced patients, 18 had one previous treatment failure, 14 had two previous treatment failures, and 10 had three or more previous treatment failures.

**Figure 2:**
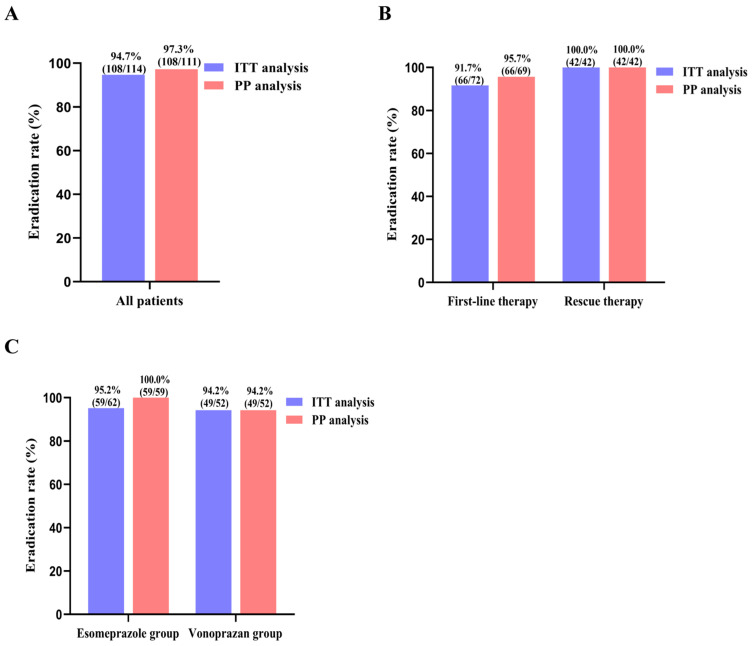
The eradication rates of minocycline-based quadruple therapy. (**A**) the overall eradcation rates; (**B**) the eradication rates among the treatment-naïve group and rescue group; (**C**) the eradication rates according to esomeprazole and vonoprazan. ITT, intention-to-treat; PP, per protocol.

The overall eradication rates were 94.7% (108/114) and 97.3% (108/111) by ITT and PP analysis, respectively (Figure 2A). The eradication rates among the treatment-naïve group were 91.7% (66/72) and 95.7% (66/69) in the ITT and PP analysis, respectively. In the rescue treatment group, an eradication rate of 100% was achieved in both the ITT and PP analysis (Figure 2B). Sub-analysis of the eradication rates according to esomeprazole and vonoprazan was also performed. Interestingly, the ITT and PP eradication rates among the 62 patients receiving EMAB regimen were 95.2% (59/62) and 100% (59/59), respectively, whereas the ITT and PP eradication rates for the 52 patients receiving VMAB regimen were both 94.2% (49/52) (Figure 2C).

Adverse events were recorded in 45.6% (52/114) of the included participants. Of note, all the adverse events were mild and moderate, and no serious adverse events were recorded. Dizziness (20, 17.5%), dark color stool (15, 13.2%), and increased bowel movement (12, 10.5%) were the commonly reported adverse events (Table 2). Good compliance was achieved in 99.1% (113/114) of the patients, except for one patient who discontinued eradication treatment due to COVID-19 infection.

## 4. Discussion

With the worldwide increase in *H. pylori* resistance to clarithromycin, levofloxacin, and metronidazole, the standard triple therapy has become obsolete [18]. The current guidelines recommend classical bismuth quadruple therapy containing tetracycline and metronidazole as the first-line or rescue therapy [2,19,20]. However, the general unavailability of tetracycline, the complicated administration of both tetracycline and metronidazole (four times daily), the common adverse effects, and subsequent poor compliance greatly affect the clinical application of the classical bismuth quadruple therapy. In the current pilot study, we explored the 14-day bismuth quadruple regimen containing minocycline and amoxicillin in both first-line and rescue therapy and demonstrated an overall satisfactory ITT eradication rate of 94.7% and an excellent PP eradication rate of 97.3%. Of note, no minocycline resistance was detected among 101 clinical *H. pylori* isolates in this study.

Antibiotic resistance is an important factor in the failure of *H. pylori* eradication. A recent systematic review revealed that the primary and secondary resistance rates to clarithromycin, levofloxacin, and metronidazole have surpassed 15% in almost all WHO regions [17]. Take China for example, the current overall mean primary resistance of *H. pylori* to clarithromycin, levofloxacin, and metronidazole was 30%, 31%, and 70%, respectively [21], causing the clarithromycin-containing or levofloxacin-containing therapy to no longer be considered appropriate for empiric use unless guided by susceptibility testing. *H. pylori*’s resistance to tetracycline and amoxicillin remained negligible (<5.0%) in most of the countries [17]. Negligible tetracycline resistance was also reported in more recent studies [14,22,23,24,25]. In our study population, using MIC > 1.0 mg/L as the resistance breakpoint for both tetracycline and minocycline, the tetracycline resistance rate was 3.0%, and no isolate was found to be resistant to minocycline, suggesting that minocycline resistance is even more rare than tetracycline. Indeed, using the same MIC > 1.0 mg/L as the resistance breakpoint, the minocycline resistance rate was reported to be <1% in recent different studies [9,10,14,25]. However, the minocycline resistance rate was reported to be >5% in three studies conducted in China [12,13,16], and a much higher MIC (>8.0 mg/L) was used as the breakpoint for minocycline resistance. The higher the MIC used for the resistance breakpoint, the lower the resistance rate would be expected. We believe the comparatively high minocycline resistance rate (>5%) reported by these three studies is likely due to inappropriate laboratory testing methods.

Early studies using minocycline-containing regimens for *H. pylori* eradication started in Japan [9] and Italy [26]. Murakami et al. from Japan explored 7-day minocycline-containing triple regimens, and reported an unacceptable cure rate of 38.5% (15/39) in first-line therapy using the combination of minocycline (100 mg twice daily) + amoxicillin (750 mg twice daily), and an even lower cure rate of 9.5% (2/21) in second-line therapy using faropenem (600 mg twice daily) + amoxicillin (750 mg twice daily), whereas a comparatively acceptable cure rate of 85% (57/67) in patients infected with metronidazole-sensitive strains in second-line therapy using minocycline (100 mg twice daily) + metronidazole (250 mg twice daily) [9]. Ierardi et al. from Italy explored a 10-day minocycline-containing bismuth quadruple regimens for second-line *H. pylori* therapy and reported a cure rate of 77.7% (21/27) and 51.9% (14/27) using the antibiotic combination of minocycline (100 mg twice daily) + rifabutin (150 mg twice daily), and the combination of minocycline (100 mg twice daily) + tinidazole (500 mg twice daily), respectively [26]. The suboptimal efficacy of the early minocycline-containing regimens in the Japanese study might be ascribed to the inadequate dose of amoxicillin or nitroimidazoles, the short duration of therapy (7 days), or the antagonistic combination of minocycline with amoxicillin or faropenem.

Recent studies using minocycline-containing bismuth quadruple regimens for *H. pylori* eradication have been mainly conducted in China [11,12,13,14,15,16,27]. The most explored antibiotic combination for the minocycline-containing bismuth quadruple regimens was minocycline plus metronidazole [11,13,14,15,16]. Using a 14-day bismuth quadruple regimen containing minocycline (100 mg bid) + metronidazole (400 mg tid) in first-line therapy, Zhang et al. reported ITT and PP eradication rates of 77.1% (91/118) and 84.3 (91/108), respectively [11]. The relatively low eradication rates can be ascribed to the low dose of metronidazole. When the minocycline was used in combination with a full dose of metronidazole (400 mg qid) in a 14-day bismuth quadruple regimen, the ITT and PP eradication rates increased to 83.4–85.5% and 91.7–92.6%, respectively [13,15,16]. In a very recent multicenter study using a 14-day bismuth quadruple regimen containing the same combination of minocycline with a full dose of metronidazole in rescue therapy, the ITT and PP eradication rates were 88.0% (162/184) and 98% (149/152) despite the high level of metronidazole resistance (88.3%) [14].

There are only two previous studies that have investigated the efficacy of a 14-day bismuth quadruple regimen containing the combination of minocycline (100 mg bid) and amoxicillin (1000 mg bid) [11,12]. The ITT and PP eradication rates for first-line therapy in the two studies were satisfactory: 85.7% (102/119) and 89.5% (102/114) [11], 87.5% (140/160) and 92.6% (137/148) [12]. The ITT and PP eradication rates for rescue therapy in one of the two studies were 82.9% (58/70) and 89.1% (57/64) [12]. Our present pilot study also investigated the efficacy of the minocycline/amoxicillin-based bismuth quadruple therapy and demonstrated high ITT and PP eradication rates (91.7% and 95.7%) in first-line therapy, and an excellent eradication rate of 100% for rescue therapy in both ITT and PP analysis. The better eradication rates achieved in the present study may be partly related to the use of vonoprazan or the double dose of PPI. The superior efficacy of vonoprazan-based or double-dose PPI-based triple therapy over traditional standard-dose PPI-based triple therapy has been previously reported with similar safety and patient compliance [28,29,30,31].

The rationale of using double dose PPI or vonoprazan in the present minocycline/amoxicillin-based bismuth quadruple regimen was to maximize the success rate of *H. pylori* eradication, which is “treating it right the first time”. As the prevalence of clarithromycin-, metronidazole-, levofloxacin-, and even multi-drug-resistant *H. pylori* infection is increasing alarmingly [17], if the initial treatment fails, the bacterium becomes resistant to more antibiotics, making the rescue treatment more difficult. However, the high cure rates of the double dose PPI or vonoprazan–amoxicillin dual therapy achieved in several recent studies [32,33,34] suggest that in the context of sustained suppression of intragastric acid by potent acid inhibitors, a single sensitive antibiotic is already enough for successful *H. pylori* eradication. Indeed, a very recent study by Gao et al. reported that a 14-day vonoprazan (20 mg bid) and tetracycline (500 mg tid for body weight < 70 kg, and 500 mg qid for body weight ≥ 70 kg) dual therapy achieved an excellent eradication of 100% (18/18) for first-line therapy, and 90.9% (40/44) for second-line therapy [35]. This prompted us to think that in the context of using vonoprazan (20 mg bid) or esomeprazole (40 mg bid), the single use of minocycline might be as effective as the bismuth quadruple regimen used in the present study. This would suggest that the component of amoxicillin (1 g bid) or even the bismuth is probably unnecessary. Future studies will need to investigate the efficacy of minocycline-based dual therapy and compare it with the efficacy of minocycline/amoxicillin-based bismuth quadruple therapy in the present study, or with the efficacy of the classical high-dose dual therapy.

The combination of amoxicillin and minocycline for *H. pylori* eradication is attractive as both drugs have excellent antibacterial effects against *H. pylori*, very low *H. pylori* resistance, sound safety profile, and wide availability. Nevertheless, there are concerns that the combination of amoxicillin and minocycline may appear antagonistic [12]. As amoxicillin is an excellent bactericidal agent, whereas minocycline is a bacteriostatic agent, their combination may reduce the bactericidal effect of amoxicillin. However, the similar combination of amoxicillin with the bacteriostatic antibiotic clarithromycin is not antagonistic and has proven to be one of the most successful antibiotic combinations for treating patients with clarithromycin-susceptible *H. pylori* infection. The antibacterial effect of minocycline in combination with amoxicillin can be further studied using the fractional inhibitory concentration index to assess their in vitro interactions.

Adverse effects and patient compliance also have a great impact on the efficacy of *H. pylori* eradication therapies. In our study, the most commonly reported adverse event was dizziness, which was consistent with those of previous studies investigating minocycline-based regimens [11,12,13,14,15,16,27]. As minocycline is more lipophilic than other tetracyclines and capable of crossing the blood–brain barrier, dizziness can be considered a specific side effect of minocycline [36]. Of note, in the present study, the occurrence of dizziness (17.5%) in our patients treated with minocycline/amoxicillin-containing bismuth quadruple therapy was significantly less common than the 44.0% of dizziness reported in patients treated with minocycline/metronidazole-containing bismuth quadruple therapy [14]. It is likely that the combination of minocycline with metronidazole significantly increased the frequency of dizziness. Of note, all the adverse events in our study were mild and moderate, and no patient discontinued treatment due to adverse events. This can be ascribed to the simple and practicable administration of this modified bismuth quadruple regimen: all drugs were given twice daily, which helped to improve the patient’s treatment compliance. The four times daily administration of full-dose metronidazole in previous minocycline-containing bismuth quadruple regimens was complicated for patients and resulted in severe adverse events, which in turn led to treatment discontinuation in more than 10% of the patients [14].

As a pilot study, there are several limitations. Firstly, the sample size was small and it was a single-center study. The excellent eradication efficacy obtained in this study warrants a further, large-sample, multicenter, and randomized controlled study to verify the efficacy of this minocycline/amoxicillin-containing bismuth quadruple regimen. For instance, future studies need to compare the efficacies of different durations (7-day, 10-day, and 14-day) of this minocycline/amoxicillin-containing bismuth quadruple regimen with the classical tetracycline/metronidazole-containing bismuth quadruple regimen, or with the high dose dual therapy (either vonoprazan-based or double dose PPI-based). Secondly, since this was a pilot study, gastroendoscopy was not recommended to the included patients treated with the minocycline/amoxicillin-containing bismuth quadruple regimen, and *H. pylori* culture and antimicrobial susceptibility were not performed for these patients. In future multicenter studies, gastroendoscopy and *H. pylori* culture/antimicrobial susceptibility will be performed to characterize antibiotic resistance profiles, not only for minocycline and tetracycline but for all other commonly used anti-*H. pylori* antibiotics. Thirdly, regarding the use of gastric acid inhibitors, both esomeprazole and vonoprazan were used in this pilot study, which is a source of heterogeneity for the minocycline/amoxicillin-based bismuth quadruple therapy regimens. As vonoprazan has recently been approved by the China National Medical Products Administration (NMPA) for *H. pylori* eradication treatment, more vonoprazan-containing therapy regimens are expected to be investigated in China.

## 5. Conclusions

Minocycline/amoxicillin-based bismuth quadruple therapy is an excellent therapy for fist-line and rescue *H. pylori* eradication treatment with good safety and excellent compliance. The two-times daily administration of both minocycline and amoxicillin makes this regimen a convenient treatment therapy for *H. pylori*-infected patients. As an excellent eradication rate of 100% was observed in patients with *H. pylori* resistant to previous treatments in this pilot study, it would be very useful to assess whether a shorter duration of treatment is able to reach the same excellent results as the 14-day duration therapy. Therefore, a well-designed prospective multicenter, and randomized controlled study is needed to confirm the efficacy of minocycline-based therapy in the future.

## Figures and Tables

**Table 1:** Patient demographic and clinical characteristics.

Variables	Patients
Gender (male/female)	60/54
Age, mean ± SD (years)	48.8 ± 11
Number of previous treatments	
0	72 (63.2%)
1	18 (15.8%)
2	14 (12.3%)
≥3	10 (8.8%)

**Table 2:** Adverse effects of minocycline-based quadruple therapy.

Variables	Patients (*n*,%)
Dizziness	20 (17.5%)
Dark color stool *	15 (13.2%)
Increased bowel movement	12 (10.5%)
Nausea	8 (7.0%)
Diarrhea	4 (3.5%)
Fatigue	6 (5.3%)
Abdominal pain	3 (2.6%)
Abdominal distension	5 (4.4%)
Belching	1 (0.9%)
Headache	1 (0.9%)

* related to the administration of bismuth, not indicating upper gastrointestinal bleeding.

## Data Availability

Data are contained within the article.

## Supplementary Materials

The following supporting information can be downloaded at: https://www.mdpi.com/article/10.3390/microorganisms12030429/s1, Table S1: Minimum inhibitory concentrations (MICs) of tetracycline and minocycline to 101 clinical *H. pylori* strains.
