# Supplementary material for: Minocycline/Amoxicillin-Based Bismuth Quadruple Therapy for Helicobacter pylori Eradication: A Pilot Study
Source: Microorganisms. 2024 Feb 20;12(3):429. doi: 10.3390/microorganisms12030429 (PMC10972041; doi:10.3390/microorganisms12030429)
Supplement: Supplementary file 1 [file microorganisms-12-00429-s001.zip › microorganisms-2802595-supplementary.pdf]

**Table S1.** Minimum inhibitory concentrations (MICs) of tetracycline and minocycline to 101 clinical *H. pylori* strains.

| No. | Tetracycline (mg/L) | Minocycline (mg/L) | No. | Tetracycline (mg/L) | Minocycline (mg/L) |
|---|---|---|---|---|---|
| 1 | 0.047 | 0.047 | 52 | 0.023 | 0.023 |
| 2 | 0.032 | 0.016 | 53 | 0.047 | 0.032 |
| 3 | 0.064 | 0.016 | 54 | 0.047 | 0.023 |
| 4 | 0.016 | 0.016 | 55 | 6 | 0.023 |
| 5 | 0.094 | 0.047 | 56 | 0.38 | 0.023 |
| 6 | 0.19 | 0.023 | 57 | 0.094 | 0.047 |
| 7 | 0.25 | 0.032 | 58 | 0.047 | 0.032 |
| 8 | 0.094 | 0.047 | 59 | 0.023 | 0.016 |
| 9 | 0.064 | 0.016 | 60 | 0.047 | 0.023 |
| 10 | 0.19 | 0.064 | 61 | 0.064 | 0.016 |
| 11 | 0.125 | 0.047 | 62 | 0.064 | 0.032 |
| 12 | 0.047 | 0.032 | 63 | 0.047 | 0.016 |
| 13 | 0.047 | 0.016 | 64 | 0.025 | 0.047 |
| 14 | 0.032 | 0.023 | 65 | 0.064 | 0.032 |
| 15 | 0.125 | 0.032 | 66 | 0.064 | 0.047 |
| 16 | 0.032 | 0.032 | 67 | 0.19 | 0.032 |
| 17 | 0.094 | 0.016 | 68 | 1.5 | 0.38 |
| 18 | 0.064 | 0.023 | 69 | 0.064 | 0.016 |
| 19 | 0.38 | 0.064 | 70 | 0.19 | 0.047 |
| 20 | 0.047 | 0.023 | 71 | 0.023 | 0.047 |
| 21 | 0.19 | 0.023 | 72 | 0.047 | 0.032 |
| 22 | 0.064 | 0.032 | 73 | 0.064 | 0.023 |
| 23 | 0.094 | 0.047 | 74 | 0.094 | 0.064 |
| 24 | 0.094 | 0.032 | 75 | 0.047 | 0.016 |
| 25 | 0.38 | 0.064 | 76 | 0.25 | 0.047 |
| 26 | 0.032 | 0.023 | 77 | 0.19 | 0.047 |
| 27 | 0.19 | 0.047 | 78 | 0.5 | 0.125 |
| 28 | 0.016 | 0.023 | 79 | 0.125 | 0.094 |
| 29 | 0.125 | 0.064 | 80 | 0.094 | 0.032 |
| 30 | 0.032 | 0.016 | 81 | 0.38 | 0.047 |
| 31 | 0.023 | 0.032 | 82 | 2 | 0.19 |
| 32 | 0.125 | 0.032 | 83 | 0.25 | 0.064 |
| 33 | 0.032 | 0.016 | 84 | 0.023 | 0.023 |
| 34 | 0.032 | 0.016 | 85 | 0.047 | 0.032 |

| | | | | | |
|---|---|---|---|---|---|
| 35 | 0.032 | 0.016 | 86 | 0.064 | 0.032 |
| 36 | 0.125 | 0.032 | 87 | 0.064 | 0.023 |
| 37 | 0.023 | 0.032 | 88 | 0.19 | 0.023 |
| 38 | 0.064 | 0.016 | 89 | 0.047 | 0.047 |
| 39 | 0.064 | 0.016 | 90 | 0.047 | 0.016 |
| 40 | 0.19 | 0.047 | 91 | 0.064 | 0.016 |
| 41 | 0.064 | 0.047 | 92 | 0.032 | 0.016 |
| 42 | 0.094 | 0.032 | 93 | 0.094 | 0.023 |
| 43 | 0.032 | 0.047 | 94 | 0.023 | 0.016 |
| 44 | 0.125 | 0.047 | 95 | 0.064 | 0.023 |
| 45 | 0.023 | 0.047 | 96 | 0.047 | 0.032 |
| 46 | 0.19 | 0.016 | 97 | 0.064 | 0.016 |
| 47 | 0.094 | 0.047 | 98 | 0.064 | 0.032 |
| 48 | 0.032 | 0.023 | 99 | 0.094 | 0.016 |
| 49 | 0.064 | 0.016 | 100 | 0.094 | 0.032 |
| 50 | 0.023 | 0.016 | 101 | 0.125 | 0.023 |
| 51 | 0.032 | 0.032 | | | |
